# Transcatheter Closure of Patent Ductus Arteriosus in Elderly Patients: Initial and One-Year Follow-Up Results—Do We Have the Proper Device?

**DOI:** 10.1155/2020/4585124

**Published:** 2020-04-29

**Authors:** Michal Galeczka, Malgorzata Szkutnik, Jacek Bialkowski, Sebastian Smerdzinski, Mateusz Knop, Adam Sukiennik, Roland Fiszer

**Affiliations:** ^1^Department of Congenital Heart Defects and Paediatric Cardiology, FMS in Zabrze, Medical University of Silesia in Katowice, Silesian Centre for Heart Diseases, Zabrze, Poland; ^2^Department of Cardiology and Internal Diseases, University Hospital No. 1, Collegium Medicum in Bydgoszcz, Nicolaus Copernicus University in Torun, Torun, Poland

## Abstract

**Objectives:**

Patent ductus arteriosus (PDA) in elderly patients is an uncommon anomaly, and the duct itself is often calcified and fragile; therefore, transcatheter closure is more difficult. The aim is to analyse periprocedural and one-year follow-up results of transcatheter closure of PDA in such patients. *Methods and results*. Retrospective analysis of 33 elective patients aged ≥55 years (median 63; 56–85; 29 women), in whom PDA was closed percutaneously between 2002 and 2018 in two tertiary centres. All but three patients were symptomatic, with most in NYHA II (*n* = 14) and III (*n* = 11) class; pulmonary hypertension (*n* = 22), arterial hypertension (*n* = 22), duct calcifications (*n* = 17), atrial fibrillation (*n* = 15), significant mitral regurgitation (*n* = 5), and decompensated renal failure (*n* = 2) were observed. Different devices were applied depending on PDA morphology; nitinol wire mesh occluders with symmetrical articulating discs have been the most used in recent years (*n* = 11). Follow-up was conducted at an outpatient clinic (28/33 patients). The procedure was successful in all patients. There was one embolisation, followed by implantation of a larger device. No major complications were noted. A small residual shunt was present in echocardiography in one patient after one year. NYHA class improved in all but two patients (with multiple comorbidities).

**Conclusions:**

Transcatheter PDA closure in elderly patients is safe and efficient with a high complete closure rate and few complications. Amplatzer duct occluder type II is an attractive device in such patients.

## 1. Introduction

Patent ductus arteriosus (PDA) is an uncommon anomaly in adult patients, as it is usually identified in childhood. Numerous pathological changes occur in adult PDA, including calcification, friability, aneurysm formation, tortuosity, and ductal shortening [1, 2]. Surgical closure of such ducts is complex, often associated with serious complications, and cardiopulmonary bypass may be inevitable [1]. With the development of coils and nitinol wire mesh duct occluders, percutaneous closure has become the method of choice in PDA treatment, also in this age group. However, the literature concerning transcatheter closure of PDA in elderly patients, especially those with a calcified duct, is scarce [3–6].

## 2. Materials and Methods

### 2.1. Patients

From October 1993 to December 2018, over 1000 consecutive patients had a PDA percutaneously closed at two tertiary centres. All 33 patients older than 55 years old (y) were included in the retrospective study (Table 1). Patients' characteristics and periprocedural and one-year observations were analysed. Clinically silent duct, more than 12 mm in wide PDA, and Eisenmenger syndrome were exclusion criteria for PDA closure. Written informed consent was obtained from all patients prior to the procedure. The study was approved by an appropriate ethics committee.

### 2.2. Transcatheter Procedure

**Figure 1:**
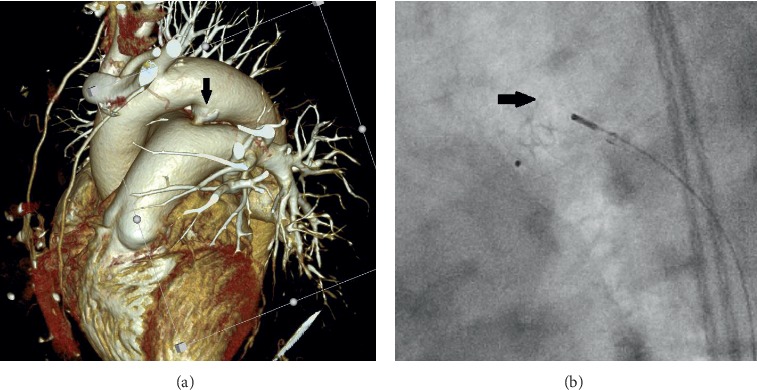
(a) Three-dimensional computed tomography reconstruction of a 5 mm PDA type A with calcifications (arrow). (b) Fluoroscopy in lateral view. 5 × 4 mm Amplatzer duct occluder type II on its delivery cable deployed on the basis of PDA aortic ampulla calcifications (arrow).

**Figure 2:**
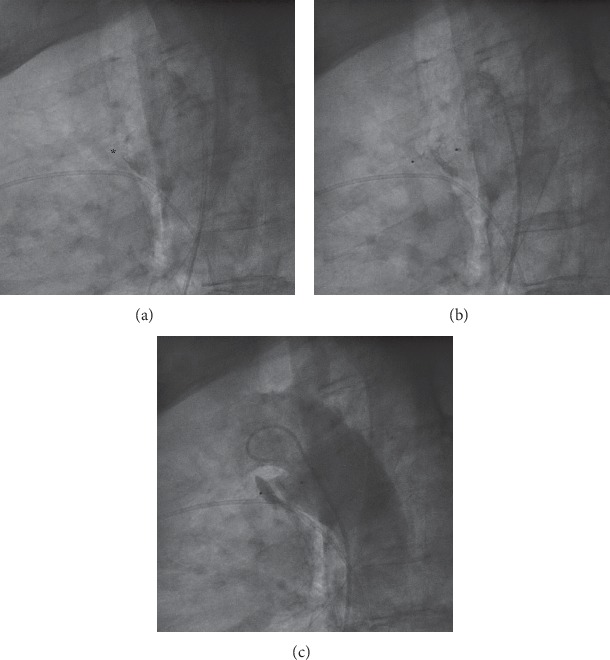
Fluoroscopy/aortography in lateral view. (a) Severely calcified 5 mm PDA type A. ^*∗*^Pulmonary end of PDA. (b, c) 6 × 4 mm Amplatzer duct occluder type II implanted from arterial approach, a small (transient) residual shunt in aortography.

**Figure 3:**
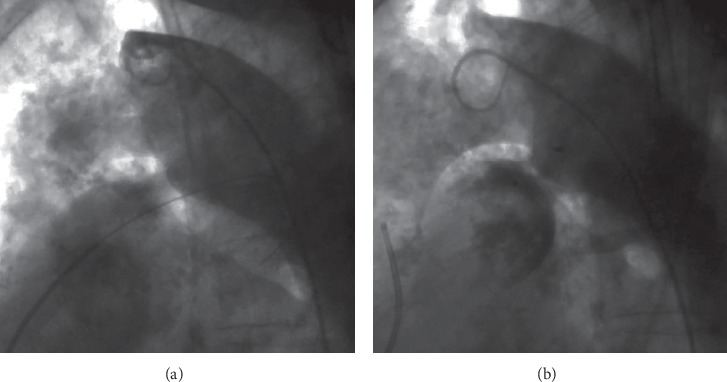
Aortography in lateral view. (a) Calcified 4 mm PDA type C in patient with a mean pulmonary artery pressure of 51 mm Hg. (b) 10 mm ventricular septal occluder implanted from arterial approach.

All procedures were performed under local anaesthesia and fluoroscopic guidance. After femoral artery (and vein) access completion (6-French sheath), intravenous heparin (50 IU/kg) and cefazolin were administered. Right and left heart pressures were measured, followed by angiography in lateral and/or right anterior oblique projections. PDA anatomy was classified as type A in 65.6% (21/32), B in 9.4% (3/32), C in 15.6% (5/32), D in two patients, and E in one patient [7]. Pronounced calcifications within PDA were observed in 51.5% patients (17/33) by fluoroscopy. In two patients with decompensated renal failure, preimplantation angiography was abandoned, and the device was selected and deployed on the basis of PDA calcifications and its measurements (Figure 1 and Video 1 in supplementary material). The median narrowest PDA diameter and length were 4.2 mm (1.5–7) and 9 mm (3–24), respectively. Static balloon calibration with an 18 mm Amplatzer sizing balloon (AGA Medical Corp., Plymouth, MN, USA) for better duct visualisation was performed in 7 patients (21.2%) and only during the beginning of our expierience in PDA closure in those patients. Recently, computed tomography has been used for this purpose and performed in 4 patients (12.1%). The median mean pulmonary artery pressure (mPAP) was 30.5 mm Hg (12–55) with a value >25 mm Hg in 66% patients. In one patient with a mPAP of 55 mm Hg (>50% of systemic pressure), the balloon occlusion test was performed before PDA closure, and mPAP decreased to 27 mm Hg. Nitinol wire mesh occluders were applied in all but two patients, and depending on their availability: duct occluder type I in 17 patients (DO I: among them 8 Amplatzer, 5 HeartR, 3 Cardi-O-Fix, and 1 Hyperion), Amplatzer duct occluder type II (ADO II: Figure 2 and Video 2 in supplementary material) in 7 patients, Amplatzer Duct Occluder II Additional Sizes (ADO II AS) in 2 patients, Amplatzer muscular VSD occluder (VSO; Figure 3) in 3 patients, Amplatzer vascular plug type II (AVP II) in 2 patients, Amplatzer septal occluder in 1 patient, as well as StarFlex device in 2 patients. As majority of ducts were type A and >3 mm in wide, DO I was our first choice until introduction of devices with symmetrical retention discs (AVP II, ADO II, and II AS). Moreover, in PDA type B atrial septal occluders and in patients with high mPAP (>50 mm Hg), VSO were generally used. DO I minimally 2 mm greater than the narrowest PDA diameter were chosen. In 24 patients, the anterograde (venous) and in 9 patients, the retrograde (arterial) delivery approach was employed. The latter devices were ADO II (*n* = 6), ADO II AS (*n* = 2), VSO (*n* = 1), and AVP II (*n* = 1). In 11/24 patients (46%), due to difficulty with anterograde crossing of PDA, the retrograde wire-assisted technique modification was applied. The snare introduced with a left Judkins catheter retrogradely through the PDA was used to catch the tip of a 0.035″ hydrophilic guidewire in the pulmonary artery and to pull it into the descending aorta. Then, the delivery system was introduced over the stiff guidewire from the venous access. Devices were released after confirmation of a stable position by control angiography. In selected patients, the PAP measurement was repeated.

Protrusion was defined as blood flow turbulence either in the descending aorta or in the pulmonary artery with a velocity >2.0 m/s by Doppler echocardiography.

### 2.3. Follow-up Protocol

Transthoracic echocardiography (TTE) was performed within 24 hours postprocedure and before discharge. Follow-up data were collected during previously scheduled visits in the outpatient clinic 1, 6, and 12 months after the procedure and included a clinical examination, electrocardiography, and TTE.

### 2.4. Statistics

All continuous variables are expressed as mean values and standard deviation or median with range as appropriate, and discrete variables are presented as percentages. Univariate analysis was performed by Student's *t*-test or Mann–Whitney test. A *p* value <0.05 was considered statistically significant. The data were analysed with Statistica 13.3 software (StatSoft Inc.).

## 3. Results

### 3.1. Patients

There were 33 patients: 29 women and 4 men with a median age of 63 y (range: 56–85). The median weight was 66 kg (45–92); 51.5% patients were overweight (17/33) and 18.2% were obese (6/33). All but three patients were symptomatic with most patients in NYHA II and III class (14 and 11 patients, respectively). Continuous murmur was present in 57.6% patients (19/33). Most patients had multiple concomitant comorbidities, predominantly arterial hypertension (*n* = 22), hypercholesterolemia (*n* = 15), coronary artery disease (*n* = 6), as well as PDA implications: atrial fibrillation (AF; *n* = 15) and moderate/severe mitral regurgitation (MR; *n* = 5). One patient had PDA surgically ligated (16 y before) and was qualified for PDA closure due to a residual shunt. All patients presented increased pulmonary vascular marking and/or enlargement of the cardiac silhouette in the chest X-ray. The left atrium was enlarged in 66.7% patients (22/33) and left ventricle in 63.6% patients (21/33) in TTE [8].

### 3.2. Transcatheter Procedure

PDA closure was successful in all patients. In one patient with low ejection fraction and with a type E duct (narrowest diameter 1.5 mm, length 9 mm), the ADO II AS 3 × 4 mm implanted from the arterial side embolised to the left pulmonary artery just after release. Then, ADO II 6 × 6 mm was implanted with success (from the same approach) and the remaining device was retrieved with a lasso. In one patient with a 4.9 mm type A duct, an unstable 8/10 mm DO I was exchanged for a 10/12 mm size. There were moderate residual shunts in 2, small in 3, and none in the remaining patients observed by control aortography. Moderate groin haematoma in 5 patients and new onset of AF which required cardioversion (after 24 hours) in one patient were the only complications. There was no protrusion of the device in any patient. Three patients with arterial hypertension needed temporal nitroglycerine infusion after PDA closure.

In patients with mPAP >35 mm Hg, in whom the pulmonary artery pressure measurement was repeated after PDA closure, the mean mPAP decreased from 41.3 to 29.3 mm Hg (*n* = 6, *p*=0.02). Median fluoroscopy and procedure time were 10 and 58 minutes (min), respectively. Fluoroscopy time was shorter in patients with the arterial vs. venous implantation approach (5.7 vs. 13.6 min; *p*=0.009). Among patients with anterograde approach, the fluoroscopy time was significantly longer in procedures with arteriovenous loop formation vs. those with a direct anterograde approach (16.8 vs. 10.9 min; *p*=0.03). Two patients with calcified duct type A and with decompensated renal failure had PDA closed with ADO II and AVP II from the arterial approach without preimplantation angiography. Both procedures were uneventful, and fluoroscopy and procedure time were shorter than median values (average 4 and 37.5 min, respectively).

TTE revealed a small residual shunt in two patients within 24 hours postprocedure (VSO and ADO II) and in only one of them before discharge (3.0%, VSO).

### 3.3. Follow-up

**Figure 4:**
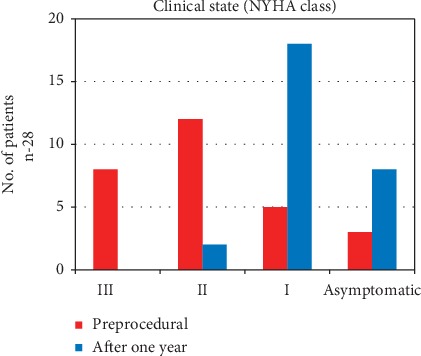
Clinical state (New York Heart Association (NYHA) class): preprocedural and after one year.

One-year follow-up was available for 84.8% patients (28/33). Either NYHA class had improved or symptoms had subsided in all but two patients (with multiple comorbidities). NYHA was improved from III to I class in 8 patients and from II to I class in 10 patients (*p* < 0.001; Figure 4). A trivial residual shunt persisted in one patient (3.0%, VSO). Both left atrium and left ventricle diastolic diameter decreased in TTE, but not significantly, from 44 to 41.4 (*p*=0.56) and from 54 to 47.9 mm (*p*=0.13), respectively (*n* = 11). There were no related complications including late embolisation, infective endocarditis, new arrhythmias, or death at any follow-up time point. One patient with severe MR and AF underwent elective ablation of AF after 3 months. Among patients with significant MR (*n* = 5), clinical state has improved after the procedure in all of them (from NYHA II or III to I class), and the left atrium diameter has decreased insignificantly (50.3 vs. 47.3 mm, *p* > 0.05).

## 4. Discussion

**Figure 5:**
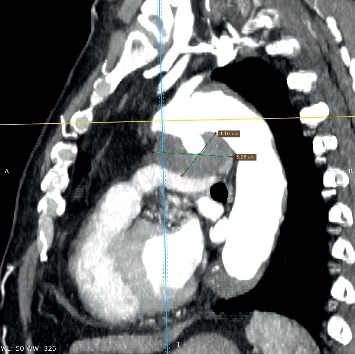
Computed tomography angiography scan of an older man with partially thrombosed PDA aneurysm (own material).

PDA in the elderly, with an estimated mortality of 1.8% per annum, should be treated to prevent bacterial endocarditis, pulmonary vascular disease, and congestive heart failure with frequent atrial flutter or/and fibrillation [9]. AF, caused by left atrium volume overload in adults with PDA, was present in as many as 45.5% of our patients, which is significantly higher than among the overall population at this age [10]. Adult PDA is frequently characterised by abnormal shape, large size, concomitant calcifications, tortuosity, or aneurysms (Figure 5). Among our population, patients with calcified duct were insignificantly older than those without calcifications (67.5 vs. 62.1 y, *p*=0.1), body mass index, and occurrence of hypercholesterolemia and arterial hypertension did not differ significantly between them (*p* > 0.05). Before the era of percutaneous treatment, most adult PDAs must have been closed using cardiopulmonary bypass with patching of the main pulmonary artery; thus, any approach by transcatheter closure is likely to be of lower risk [11]. However, surgical treatment in adults remains the method of choice in wide, deformed ducts, especially when associated with concomitant operative heart disease [12].

To date, the literature concerning percutaneous PDA treatment in elderly patients is scarce, but the results with the Rashkind double umbrella (no longer used) and DO I are promising [4–6, 13]. According to the ductal anatomy, various off-label devices have been also reported such as VSO and stent grafts [14, 15]. The fragile and often calcified PDA in adults differ from those seen in childhood, making transcatheter closure technically much more demanding than in children and with longer fluoroscopy time [16].

We report 33 elective patients older than 55 y with successful PDA transcatheter closure using different devices. Duct static balloon calibration was performed in 21.2% of patients since high flow and large ducts diameter in adults lead to suboptimal angiography definition [17]. This diagnostic method was adopted only during the beggining of our expierience in PDA closure in those patients. Computed tomography has been performed recently in special situations (12.1%) before PDA closure for better duct anatomy delineation, which has replaced balloon calibration.

In 7/13 patients with calcified ducts (54%) and in 4/11 patients with noncalcified ducts (36%), anterograde PDA crossing was difficult and the adopted retrograde wire-assisted technique was applied. This is a well-described difficulty among elderly patients with PDA [3, 18] and, as in our cohort, more frequently appears among calcified ducts.

Various devices have been adopted in our practice, depending on duct morphology and their availability: from StarFlex, introduced in the 1990s, to the more recently introduced ADO II and ADO II AS. Nowadays, all PDA types can be treated with a wide range of armamentarium. To our best knowledge, this is the first report covering so many different types of implants applied in PDA in the elderly.

DO I introduction in the 1990s greatly changed the method of PDA treatment. However, in elderly patients, crossing the fragile and calcified duct with a large and stiff sheath is a major disadvantage. Since the introduction of ADO II into our practice, it has replaced DO I and has become our first choice of device in adult PDA. ADO II, designed for the treatment of ducts less than 5.5 mm in wide and less than 12 mm long, was used in 7 of our patients (with PDAs type A, C, D, and E). Relatively much larger, articulating, and symmetrical discs in comparison to the waist ensure stable positioning of the device. The elastic properties of ADO II discs enable their accurate adjustment to the margins of the calcified ducts. Despite this fact, as ADO II does not have a prothrombotic polyester cloth inside, only in one patient, a small residual leak had been observed after 24 hours in TTE, but it had yielded until discharge. ADO II can be implanted from the arterial approach, which excludes the need to establish an arteriovenous loop; moreover, it requires only a 4- or 5-French loading sheath, which makes it attractive in elderly patients with calcified and fragile ducts. AVP II, which is a device of similar design to ADO II, was used before ADO II implementation with positive results. Moreover, it can be applied in ducts bigger than those suitable for ADO II. Nitinol wire mesh devices with articulating symmetrical retention discs and a stenting central waist, such as ADO II and II AS and AVP II, seem to be attractive for the transcatheter closure of elderly PDA.

Special difficulties are related to the considerably rare PDA type B anatomy. In this morphology, two StarFlex devices and one septal occluder were used with good results, which has been reported before [19].

Residual shunts and haemolysis are more frequently found in percutaneously closed calcified ducts [3]. However, a small residual shunt in only one patient at follow-up (VSO, calcified duct) and no case of haemolysis were found in our analysis.

Transcatheter PDA closure can decrease the left ventricle volume in adult patients with severe MR [20]. We found NYHA class improvement in all patients with more than moderate MR, in whom PDA was closed.

As an adult PDA is a rare finding, the individual approach for its transcatheter closure should be underlined, especially in heavily calcified and, therefore, fragile ducts. However, we find ADO II in ≤5.5 mm ducts and AVP II in ≥6 mm ducts useful in this group of patients, particularly in PDA types C, D, and E.

### 4.1. Limitations

A small population from two centres and the retrospective nature of this report are the main limitations.

## 5. Conclusion

Transcatheter closure of PDA in elderly patients, also with calcified ducts, is a safe and efficient method of treatment with a high complete closure rate and low number of complications. Difficulty in crossing calcified ducts and the need for arteriovenous loop formation increase the fluoroscopy time. ADO II, which can be deployed from the arterial side, is an attractive device in such patients.

## Figures and Tables

**Table 1:** Clinical and procedural data of elderly patients aged >55 years old, in whom PDA was closed percutaneously.

No.	Age (y)	Weight (kg)	NYHA class before	PDA type	Calcifications (0/1)	Narrowest PDA diameter (mm)	PDA length (mm)	Implant type	Implant size (mm)	Implantation route (v/a)	Complications/others	Fluoroscopy time (min)	Procedure time (min)	Residual shunt in echocardiography after 24 hours/one year	NYHA class after one year
1	62	90	1	A	0	6	8	DO I	8/10	v	Nitroglycerine infusion	14	60	None	No symptoms
2	62	64	—	B	1	5	5	StarFlex	23	v		28	135	None	No symptoms
3	58	62	2	C	0	4	4	DO I	6/8	v		16	90	None	1
4	56	60	2	A	0	5.7	24	DO I	10/12	v	Groin haematoma	11	55	None	—
5	56	84	3	A	1	7	8	VSO	12	v	Groin haematoma	14	145	None	1
6	56	68	3	Postsurgery	1	5	20	DO I	8/10	v		15	58	None	1
7	75	77	2	B	1	6	3	StarFlex	23	v		29	95	None	—
8	85	50	3	A	1	3.2	16	DO I	8/10	v		12	90	None	—
9	65	85	2	A	0	6.7	12	DO I	10/12	v		23	80	None	1
10	58	78	3	C	0	5.2	—	DO I	8/10	v	Groin haematoma, nitroglycerine infusion	13	50	None	—
11	63	48	2	A	1	5.5	—	VSO	8	v	AF cardioversion	6	45	None	1
12	73	56	3	A	1	4	7	DO I	8/10	v		11	50	None	1
13	72	50	3	B	1	6	4	ASO	8	v		28	100	None	—
14	58	82	2	A	1	4	—	DO I	8/10	v		6	70	None	1
15	59	64	2	A	0	4.9	12	DO I	10/12	v	8/10 unstable	15	60	None	1
16	56	63	2	A	1	4.2	10	DO I	10/12	v		22	75	None	1
17	58	45	3	A	0	5	—	DO I	8/10	v		10	50	None	1
18	57	81	2	A	0	3.5	9	DO I	8/10	v		4	45	None	1
19	76	70	3	C	1	3.5	—	DO I	8/10	v		8	43	None	1
20	64	76	3	A	1	3.8	—	DO I	10/12	v	Groin haematoma	21	60	None	1
21	64	57	3	C	1	4	7	VSO	10	a		31	80	Small/small	1
22	59	80	2	E	0	1.5	9	ADO II AS	6 × 6	a	ADO II AS embolisation to pulmonary artery, percutaneous removal; ADO II 6 × 6 mm implanted successfully	9	48	None	2
23	74	71	2	A	1	4	—	DO I	10/12	v		6	45	None	1
24	69	64	1	D	0	3	15	AVP II	10	v		4	50	None	No symptoms
25	74	66	1	A	0	4	14	DO I	10/12	v	Groin haematoma	10	75	None	No symptoms
26	56	60	—	A	0	3	—	ADO II	4 × 4	a		4	14	None	No symptoms
27	70	70	1	A	0	3.2	10	ADO II	6 × 4	v		4	45	None	No symptoms
28	56	55	—	A	0	2.5	7.5	ADO II AS	5 × 6	a		3	15	None	No symptoms
29	77	74	2	A	1	5	6	ADO II	6 × 4	a	Nitroglycerine infusion	9	90	Small/none	1
30	60	66	2	C	0	3.5	7	ADO II	5 × 6	a		6	50	None	2
31	66	92	3	A	1	7	5	AVP II	10	a		4	40	None	1
32	64	80	1	D	0	5	12	ADO II	3 × 4	a		7	65	None	No symptoms
33	72	55	2	A	1	5	9	ADO II	5 × 4	a		4	35	None	1

ADO II, Amplatzer duct occluder type II; ADO II AS, Amplatzer duct occluder type II additional sizes; ASO, Amplatzer atrial septal occluder; AVP II, Amplatzer vascular plug type II a, arterial; AF, atrial fibrillation; DO I, duct occluder type I; f, female; m, male; MR, mitral regurgitation; VSO, Amplatzer muscular VSD occluder; v, venous; y, years; —, no data.

## Data Availability

The data used to support the findings of this study are available from the corresponding author upon request.

## Supplementary Materials

Supplementary Materials Video 1: fluoroscopy in lateral view. (A) 5 mm PDA type A closed with a 5 × 4 mm Amplatzer duct occluder type II in a patient with decompensated renal failure, no preimplantation aortography, and occluder deployed on the basis of calcifications. (B) Control contrast injection through loading sheath, no residual shunt. Video 2: fluoroscopy/aortography in lateral view. (A) 5 mm PDA type D. (B) Deployment of a 6 × 6 mm Amplatzer duct occluder type II. (C) Control aortography with a trivial residual shunt.
